# In Vitro Effect of Sodium Fluoride on Malondialdehyde Concentration and on Superoxide Dismutase, Catalase, and Glutathione Peroxidase in Human Erythrocytes

**DOI:** 10.1155/2013/864718

**Published:** 2013-09-24

**Authors:** José Gutiérrez-Salinas, Liliana García-Ortíz, José A. Morales González, Sergio Hernández-Rodríguez, Sotero Ramírez-García, Norma R. Núñez-Ramos, Eduardo Madrigal-Santillán

**Affiliations:** ^1^Laboratory of Biochemistry and Experimental Medicine, Biomedical Research Division, National Medical Center “20 de Noviembre,” ISSSTE, Sn. Lorenzo No. 502, 2° piso, Col. Del Valle, 03100 México, DF, Mexico; ^2^División of Genomic Medicine, National Medical Center “20 de Noviembre,” ISSSTE, Sn. Lorenzo No. 502, 2° piso, Col. Del Valle, 03100 México, DF, Mexico; ^3^Laboratorio Medicina de Conservación, Escuela Superior de Medicina, Instituto Politécnico Nacional, Plan de San Luis y Díaz Mirón, Col. Casco de Santo Tomás, Del. Miguel Hidalgo, 11340 México, DF, Mexico; ^4^Uromédica OSF, Calle Uxmal 422, Colonia Vértiz Narvarte, 03020 México, DF, Mexico

## Abstract

The aim of this paper was to describe the in vitro effect of sodium fluoride (NaF) on the specific activity of the major erythrocyte antioxidant enzymes, as well as on the membrane malondialdehyde concentration, as indicators of oxidative stress. For this purpose, human erythrocytes were incubated with NaF (0, 7, 28, 56, and 100 **μ**g/mL) or NaF (100 **μ**g/mL) + vitamin E (1, 2.5, 5 and 10 **μ**g/mL). The malondialdehyde (MDA) concentration on the surface of the erythrocytes was determined, as were the enzymatic activities of superoxide dismutase (SOD), catalase (CAT), and glutathione peroxidase (GlPx). Our results demonstrated that erythrocytes incubated with increasing NaF concentrations had an increased MDA concentration, along with decreased activity of antioxidant enzymes. The presence of vitamin E partially reversed the toxic effects of NaF on erythrocytes. These findings suggest that NaF induces oxidative stress in erythrocytes in vitro, and this stress is partially reversed by the presence of vitamin E.

## 1. Introduction

Fluoride is a halide ion that is abundant on earth and in humans and is important for the proper development of bones and teeth. However, an excess of fluoride in the body can cause fluorosis, which may occur in an acute or chronic form [1–3]. Fluorosis is a serious public health problem, especially in developing countries, where the main source of intoxication is drinking water, which may exceed a fluoride concentration of 1 ppm [1, 2]. Fluorosis in humans causes alterations in the musculoskeletal, nervous, digestive, and hematopoietic systems [1–3].

In vertebrates (including humans), hematologic disorders are the most commonly found pathologies in subjects with fluorosis, with the following being the most important: (a) hypochromic anemia, (b) variation in the size and shape of erythrocytes, (c) presence of Heinz bodies, (d) eosinophilic leukocytosis and lymphopenia, (e) increase in the amount of methemoglobin, and (f) alterations in hematocrit. Additionally, several biochemical and structural alterations have been described in erythrocytes exposed to fluoride compounds in vitro [4–10].

Oxidative stress is a recognized mechanism of damage caused by exposure to fluoride. Oxidative stress in response to fluoride has been observed in different types of cells and tissues experimentally exposed in vitro or in vivo, as well as in the tissues of animals and people living in endemically fluoridated areas [10–15].

Oxidative stress is characterized by an excess of reactive oxygen species (ROS) that react with the main cellular macromolecules, causing alterations in cellular homeostasis [16, 17]. The main damage caused by ROS is lipid peroxidation of the polyunsaturated fatty acids of cellular membranes, from which malondialdehyde (MDA) is produced as a product. Moreover, the activity of the main antioxidant enzymes, such as superoxide dismutase (SOD), catalase (CAT) and glutathione peroxidase (GlPx), is also affected [16, 17].

The most important antioxidant systems in erythrocytes are the SOD, CAT, and GlPx enzymes, as well as oxidized/reduced glutathione and vitamin E (Vit-E); Vit-E is the main antioxidant defense of cell membranes [16–19].

As mentioned above, fluoride poisoning causes several types of alterations in erythrocytes. However, the physiopathological mechanism by which these alterations occur is not fully understood. Nevertheless, it is believed that ROS production may be one of the mechanisms of damage caused by fluoride because it has been reported that these compounds inhibit antioxidant enzyme activity and increase MDA concentration in various experimental models [14, 15]. In vitro studies evaluating the antioxidant systems of erythrocytes exposed to fluoride are scarce. The aim of this study was to determine the effect of sodium fluoride (NaF, as a source of fluoride) on the enzymatic activity of SOD, CAT, and GlPx and on MDA concentration in erythrocytes exposed to this toxin in vitro. Furthermore, we aimed to evaluate the antioxidant role of Vit-E against the possible toxic effects of fluoride.

## 2. Materials and Methods

### 2.1. Sampling

After an informed consent agreement was signed, a single blood sample was taken from healthy male subjects (*n* = 5; 25 to 30 y.o.) by venipuncture, using a Vacutainer system in tubes with anticoagulant (citrate). The samples were centrifuged at 500 g for 15 min. to remove plasma and buffy coat. The cells were then washed three times by centrifuging with cold phosphate buffered saline (PBS). The packed erythrocytes were then resuspended in PBS to obtain 50% hematocrit and used for incubation [20, 21].

All procedures were performed in accordance with the general rules and procedures approved by our institution's Ethics and Investigation Committee, who approved this study protocol.

### 2.2. Erythrocyte Treatment

Erythrocytes samples were incubated in duplicate according to previously established protocols [20]. Briefly, we proceeded as follows: 0.25 mL of packed erythrocytes was placed in test tubes, and 2.75 mL of PBS was added. This solution was incubated for 10 minutes at 37°C under constant stirring. After this adaptation time, the cells were divided into the following study groups: group A: 10 *μ*L of NaF solution to a final concentration of 7, 28, 56 and 100 *μ*g/mL and, group B: 10 *μ*L of NaF solution to a final concentration of 100 *μ*g/mL, supplemented with Vit-E at different concentrations (1, 2.5, 5 and 10 *μ*L/mL). Vit-E was dissolved in 0.05% ethanol in PBS; preliminary experiments showed that there was no apparent effect of the ethanol. NaF and Vit-E concentrations were chosen in accordance with previous reports [22–24]. For each treatment, five independent treatments were performed. As a control group, erythrocytes incubated under the same experimental conditions but without the presence of NaF or Vit-E were used.

After the erythrocytes were incubated for 3 hours at 37°C under constant stirring, the samples were centrifuged at 800 rpm for 5 minutes and washed three times with cold PBS. Erythrocytes were then used to isolate cytosol and cell membranes.

### 2.3. Cytosol and Cell Membrane Isolation

To obtain the cytosol and cell membranes, erythrocytes were lysed using a hypotonic phosphate buffer (5P8; 5 mM phosphate buffer solution, pH 8) [21]. Briefly, we proceeded as follows: one aliquot of erythrocytes was mixed with five aliquots of 5P8 solution under mild stirring for 15 minutes at 4°C. The samples were then centrifuged at 10,000 rpm for 15 minutes, and the supernatant (cytosol) was separated from the pellet and stored until further use. The pellet was washed with 5P8 at 10,000 rpm until a smooth precipitate was obtained. The final precipitate (cell membranes) was resuspended in 200–250 *μ*L of cold PBS and stored until further use. Total protein concentration of the membranes was determined according to Lowry technique [25], using bovine serum albumin as a standard.

### 2.4. Malondialdehyde (MDA) Determination

MDA concentration in erythrocyte membranes was determined using a colorimetric commercial kit (TBARS Assay Kit, Cayman Chemical Co., Ann Arbor, MI, USA) following the manufacturer's protocol. MDA determination was based on the spectrophotometric detection at 530 nm (using a Jenway 6300 spectrophotometer, Cielo Vista, CA, USA) of the thiobarbituric acid-MDA adduct formed when heated under acidic conditions, according to the method reported by Yagi [26]. MDA determination (by triplicate) was performed with 300 mg of total membrane protein, and the concentration was expressed in units of nmol/mg protein.

### 2.5. Antioxidant Enzyme Activity Determination

To determine the activity of the antioxidant enzymes SOD, CAT, and GlPx in the cytosol of erythrocytes, we first eliminated the excess hemoglobin using the method reported by J. V. Bannister and W. H. Bannister [27]. A sample of cytosol was mixed (v/v) with an ethanol/chloroform solution (5/3, v/v) under constant stirring for 10 minutes. A 1/5 volume of isotonic NaCl was then added under constant stirring. The resulting solution was centrifuged at 3000 rpm for 60 minutes to recover the hemoglobin-free supernatant (HbFS) to determine the activity of the enzymes. 

The activity of SOD, CAT, and GlPx was determined in HbFS samples (by triplicate each one) using commercial kits (Superoxide Dismutase Assay Kit; Catalase Assay Kit; Glutathione Peroxidase Assay Kit; Cayman Chemical Co.) following the manufacturer's protocol for each kit. Spectrophotometric analyses were performed using a Jenway 3600 spectrophotometer, and the activity of each enzyme was reported as U/g Hb (SOD) or *μ*mol/min/g Hb (CAT and GlPx).

### 2.6. Total Hemoglobin Determination

The concentration of total hemoglobin was determined in cytosol samples using a conventional method with Drabkin's reagent, and a standard curve is read at 540 nm in a spectrophotometer [28]. 

### 2.7. Statistical Analysis

The results are expressed as the mean ± standard deviation (S.D.) and were analyzed using GraphPad Prism V 4.00 Statistical Software (GraphPad Software, San Diego, CA, USA). The results were evaluated using Student's *t*-test taking *P* < 0.05 as statistically significant. 

## 3. Results

### 3.1. The Effect of NaF on the Concentration of MDA in the Membrane of Erythrocytes

**Figure 1:**
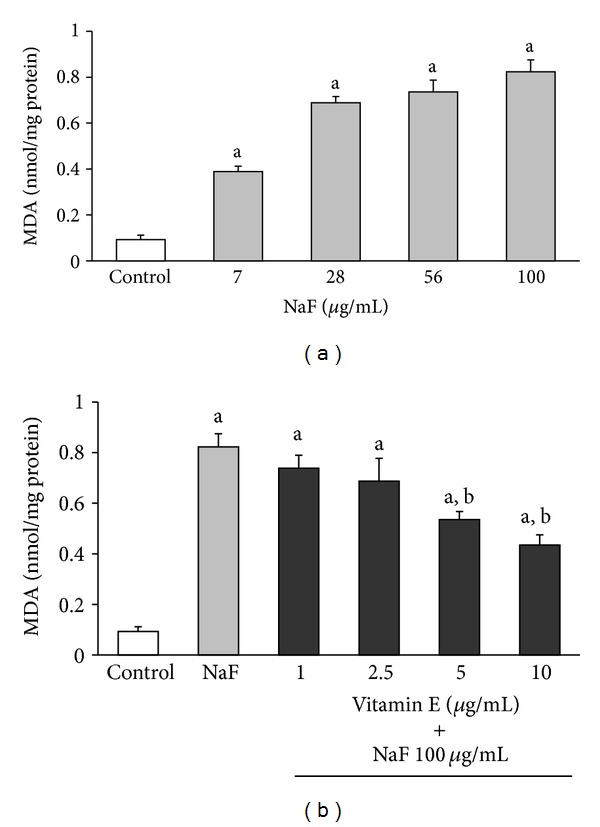
MDA concentration in the membrane of erythrocytes incubated 3 hours with increasing concentrations of sodium fluoride (NaF) (a) or incubated with 100 *μ*g/mL NaF or 100 *μ*g/mL NaF with Vit-E in increasing concentrations (b). The results are expressed as the mean ± standard deviation of five independent experiments, taking two samples per experiment and performing the determinations in triplicate. ^a^ *P* < 0.05 compared with the control group; ^b^ *P* < 0.05 compared with the group incubated with only 100 *μ*g/mL NaF.

As shown in Figure 1(a), there was a statistically significant increase in MDA concentration on the membrane of the erythrocytes exposed to increasing concentrations of NaF compared with the control. The mean MDA concentration in the control group was 0.093 ± 0.019 nmol/mg protein, whereas, with the lowest NaF concentration (7 *μ*g/mL), there was a statistically significant four-fold increase in MDA concentration (0.389 ± 0.05 nmol/mg protein; *P* < 0.005). This concentration increased up to ninefold (0.823 ± 0.072 nmol/mg protein; *P* < 0.005) with the highest NaF concentration (100 *μ*g/mL). 

Because the greatest increase in MDA concentration was obtained with 100 *μ*g/mL NaF, this concentration of the toxin was used to incubate the erythrocytes to determine the effect that increasing concentrations of Vit-E (1, 2.5, 5, and 10 *μ*g/mL) had on MDA production, as shown in Figure 1(b).

The incubation of erythrocytes with 100 *μ*g/mL NaF alone with 1 or 2.5 *μ*g/mL Vit-E had no effect on the MDA concentration produced by NaF. However, incubation with 5 and 10 *μ*g/mL Vit-E produced a statistically significant (*P* < 0.05) decrease of 35% (0.535 ± 0.0318 nmol/mg protein) and 47% (0.435 ± 0.04 nmol/mg protein) in MDA concentration, respectively, in comparison with the group incubated with 100 *μ*g/mL NaF (MDA = 0.836 ± 0.064 nmol/mg protein). However, the decrease in MDA concentration produced by the presence of Vit-E was still 4-5 times higher than that of the control group (MDA control = 0.089 ± 0.014 nmol/mg protein, Figure 1(b)).

### 3.2. Effect of NaF and Vit-E on the Activity of Antioxidant Enzymes

**Figure 2:**
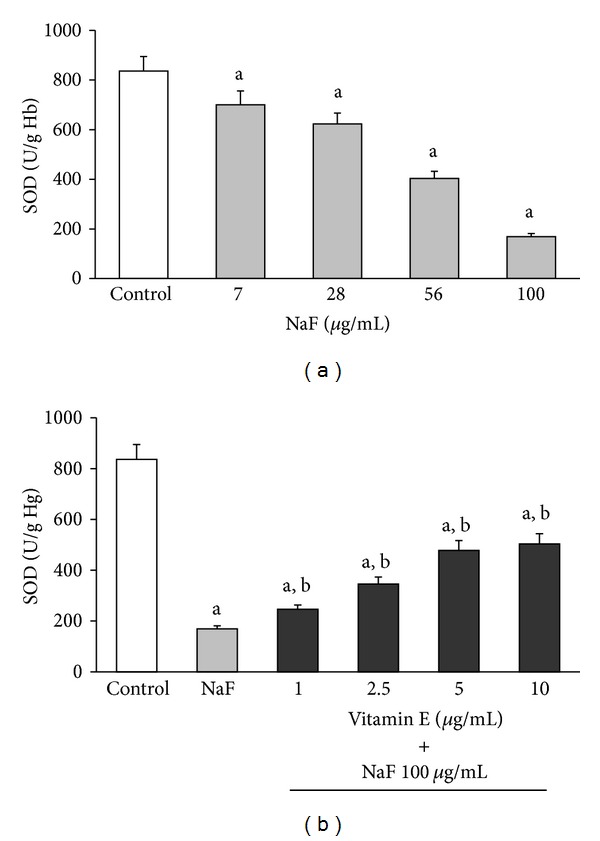
SOD activity in erythrocytes incubated in vitro for 3 hours with increasing concentrations of NaF (a) or incubated with 100 *μ*g/mL NaF or 100 *μ*g/mL NaF with Vit-E in increasing concentrations (b). The results are expressed as the mean ± standard deviation of five independent experiments, taking two samples per experiment and performing the determinations in triplicate. ^a^ *P* < 0.05 compared with the control group; ^b^ *P* < 0.05 compared with the group incubated with only 100 *μ*g/mL NaF.

**Figure 3:**
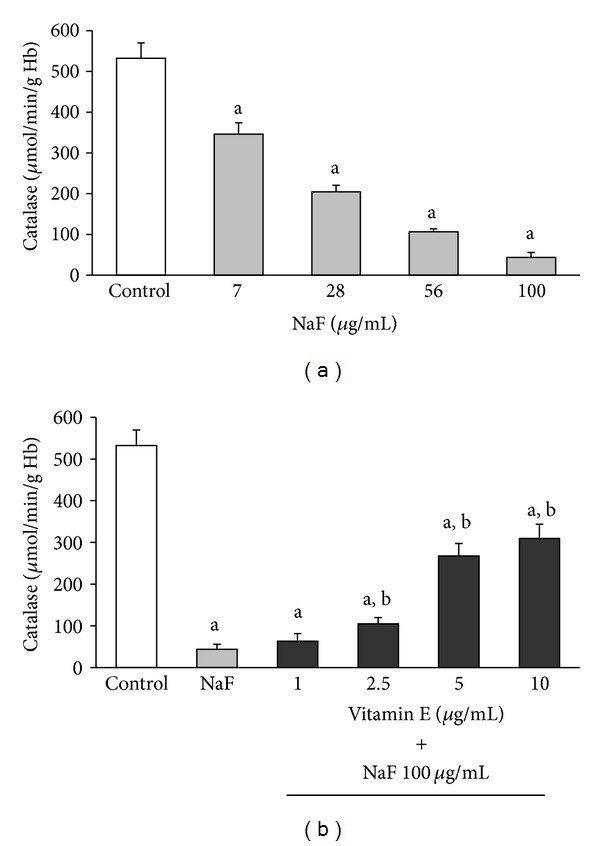
CAT activity in erythrocytes incubated under the same conditions described in Figure 2. The results are expressed as the mean ± standard deviation of five independent experiments, taking two samples per experiment and performing the determinations in triplicate. ^a^ *P* < 0.05 compared with the control group; ^b^ *P* < 0.05 compared with the group incubated with only 100 *μ*g/mL NaF.

**Figure 4:**
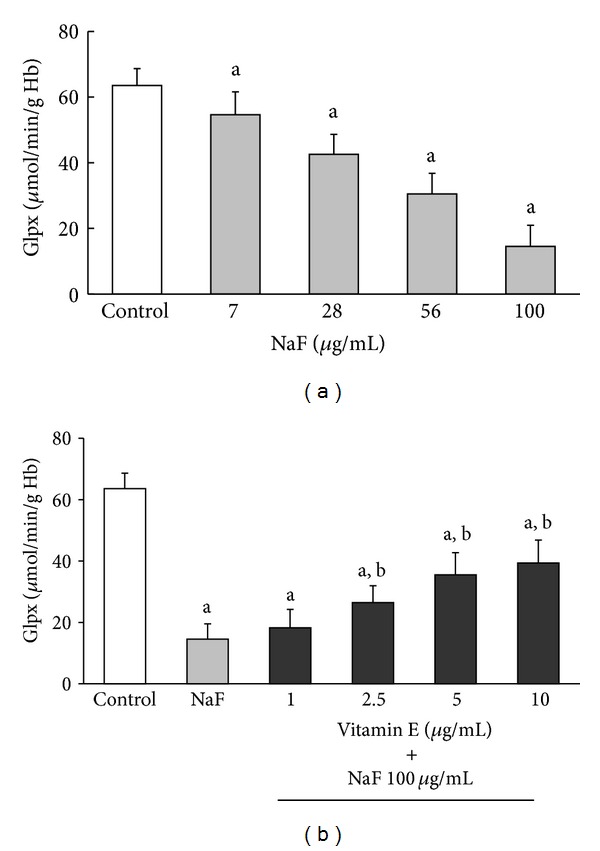
GlPx activity in erythrocytes incubated under the same conditions described in Figure 2. The results are expressed as the mean ± standard deviation of five independent experiments, taking two samples per experiment and performing the determinations in triplicate. ^a^ *P* < 0.05 compared with the control group; ^b^ *P* < 0.05 compared with the group incubated with only 100 *μ*g/mL NaF.

The activities of SOD, CAT, and GlPx in erythrocytes incubated with increasing concentrations of NaF or with 100 *μ*g/mL NaF + different concentrations of Vit-E are shown in Figures 2, 3, and 4, respectively.

The incubation of erythrocytes with increasing NaF concentrations produced a statistically significant decrease in the activity of all enzymes. This inhibition was obtained from the lowest NaF concentration (7 *μ*g/mL) and reached its maximum with the highest concentration (100 *μ*g/mL) of NaF (Section (a) of Figures 2 to 4). However, the inhibitory effect that the highest concentration of NaF (100 *μ*g/mL) had on the activity of the enzymes was partially counteracted when the cells were incubated with increasing concentrations of Vit-E (Section (b) of Figures 2 to 4). 

The activity of SOD was inhibited by 16.3% compared with the control when the erythrocytes were incubated with 7 *μ*g/mL NaF (700 ± 50 U/g Hb versus 836.25 ± 58.53 U/g Hb; *P* < 0.05). This inhibition reached 79.75% (169.36 ± 38.2 U/g Hb) with a concentration of 100 *μ*g/mL NaF (Figure 2(a)).

Moreover, when the erythrocytes were incubated with 100 *μ*g/mL NaF along with increasing concentrations of Vit-E, there was a partial reversal of the inhibitory effect of NaF, which suggested an increase in SOD activity due to the effect of Vit-E (Figure 2(b)).

Thus, erythrocytes incubated with only 100 *μ*g/mL NaF showed an average of 172.41 ± 38.2 U/g Hg SOD activity. This represents an inhibition of 79.2% (*P* < 0.05) compared with the control, which showed an average of 828.32 ± 48.33 U/g Hg SOD activity (Figure 2(b)). However, when erythrocytes were incubated with 100 *μ*g/mL NaF and 1 *μ*g/mL Vit-E, the average SOD activity was 245.89 ± 45 U/g Hg, representing 29.7% of the activity of the control. However, this value represents a statistically significant increase (*P* < 0.05) of 42.61% compared with the activity of this enzyme when the erythrocytes were incubated with only 100 *μ*g/mL NaF. The increase in SOD activity due to the presence of Vit-E (10 *μ*g/mL) was up to 60.8% (503.49 ± 52 U/g Hg) compared with the control. This represents a significant increase (*P* < 0.05) of 192.03% compared with the activity this enzyme shows in erythrocytes incubated with only 100 *μ*g/mL NaF (Figure 2(b)).

Similar to SOD, the activities of CAT and GlPx were inhibited when the erythrocytes were incubated in increasing NaF concentrations (Figures 3(a) and 4(a), resp.).

The mean activity of CAT in erythrocytes incubated under standard conditions (control) was 532.43 ± 37.27 *μ*mol/min/g Hg. This activity decreased significantly (*P* < 0.05) to 65.02% (346.2 ± 38.3 *μ*mol/min/g Hg) of the control with an NaF concentration of 7 *μ*g/mL and decreased up to 91.81% (43.59 ± 25.2 *μ*mol/min/g Hg) with a concentration of 100 *μ*g/mL NaF (Figure 3(a)). Furthermore, with a concentration of 2.5 *μ*g/mL Vit-E in the culture media of erythrocytes exposed to 100 *μ*g/mL NaF, a significant increase (*P* < 0.05) in CAT activity was observed. The increase in CAT activity was higher with increasing Vit-E concentrations, reaching up to 61.94% of the activity of the control with a concentration of 10 *μ*g/mL Vit-E. This represents a 7.52-fold increase in the activity of this enzyme compared with the activity in erythrocytes incubated with only 100 *μ*g/mL NaF (41.18 ± 22.31 *μ*mol/min/g Hg versus 309.7 ± 42.2 *μ*mol/min/g Hg; *P* < 0.05, (Figure 3(b)).

As with SOD and CAT, GlPx showed a decrease in its activity with increasing NaF concentrations. This inhibition was up to 77% (14.56 ± 6.4 *μ*mol/min/g Hg) of the control (63.56 ± 5.08 *μ*mol/min/g Hg) with the highest NaF concentration (100 *μ*g/mL) (Figure 4(a)). The presence of increasing concentrations of Vit-E in erythrocytes exposed to 100 *μ*g/mL NaF significantly increased the activity of GlPx from 13.85 ± 5.1 *μ*mol/min/g Hg (only with 100 *μ*g/mL NaF) to 39.37 ± 7.5 *μ*mol/min/g Hg (100 *μ*g/mL NaF + 10 *μ*g/mL Vit-E). This represents 61.94% of the activity of the control (Figure 4(b)).

## 4. Discussion

Fluoride is a very abundant ion in nature, where it only exists combined with other elements in fluoride compounds. The main source of fluoride for humans is the intake of water from underground aquifers contaminated with this element. The accumulation of fluoride in an organism causes fluorosis, which is manifested by damage in bone tissue as well as damage in several soft tissues and cell types such as muscle, liver, nervous system, and blood [1–3]. In areas with fluorosis, frequent hematologic alterations have been described. In vertebrates with fluorosis, hypochromic anemia, alterations in erythrocyte structure, and other hematologic alterations have been observed [3–9]. Moreover, in vitro experiments showed that alterations in metabolism as well as structural damage occur in erythrocytes exposed to fluoride compounds [10, 29].

Studies with experimental models in vivo and in vitro showed that in different tissues and cells, fluoride induces an excess of ROS production. Fluoride also decreases the biological activity of major antioxidant enzymes such as SOD, CAT, and GlPx, which play an important role in ROS elimination [11–15, 30]. However, although the mechanism by which fluoride causes these effects is not fully understood, it is thought that the generation of oxidative stress is an important part of the pathological damage mechanism induced by fluoride [11–14, 30]. Oxidative stress is an imbalance between ROS generation and the antioxidant systems. This imbalance results in damage to the major macromolecules of erythrocytes, membrane lipid peroxidation, and alterations in intermediate metabolism and hemoglobin properties [8, 9, 31–37]. In this study, we found that an in vitro increase in NaF concentration caused a corresponding increase in MDA concentration as well as decreases in the activities of SOD, CAT, and GlPx. Moreover, the presence of Vit-E in the culture media significantly inhibited the damage caused by NaF and was reflected in a decrease in MDA concentration (Figure 1) and a considerable increase in the activity of antioxidant enzymes (Figures 2 to 4).

Reports from in vitro and in vivo experiments, as well as data obtained from patients with fluorosis, indicate that NaF is an oxidative stress-causing agent. NaF increases ROS, which in turn increases MDA, as well as decreasing the activity of the major antioxidant enzymes such as SOD, CAT, and GlPx [11–15, 38].

The exact mechanism by which NaF produces these changes to the enzymatic systems associated with intermediate metabolism of the erythrocyte is not entirely known. However, it has been proposed that NaF causes these effects by interfering directly or indirectly with the main metabolic pathways of the cell. This results in an increase of ROS production and MDA concentration, caused by an alteration in the antioxidant enzymatic (e.g., SOD, CAT, and GlPx) and nonenzymatic (e.g., glutathione, urea, and Vit-E) defense systems [1–3, 11–15, 38].

A probable direct mechanism by which NaF alters the activity of antioxidant enzymes is competitive substrate inhibition. This is due to the structural similarity between NaF and some of the known substrates for oxidoreductases and ion cotransporters present in cell membranes. Indirectly, another possible effect of NaF is inducing protein denaturation because of the effect of oxidative stress it causes in cells [1–3, 6, 10–15, 18, 29, 30, 38].

Our results showed an increase in MDA concentration in the erythrocyte membrane accompanied by an alteration in the activity of antioxidant enzymes, which revealed the presence of oxidative stress in the erythrocyte caused by NaF [16–19, 31, 32]. The observed damage in the erythrocyte membrane was partially prevented by the presence of Vit-E (a well-known antioxidant commonly used in in vitro and in vivo experiments), which can act as a “trap” (scavenger) for ROS, reducing the damage they cause [24, 35, 39, 40]. Vit-E concentrations of 5 and 10 *μ*g/mL (reported as effective in decreasing ROS damage in vitro [24, 35, 39, 40]) were more effective in partially reducing the damage caused to the erythrocyte membranes by NaF.

Furthermore, our results showed that NaF inhibited the activity of SOD, CAT, and GlPx in erythrocytes exposed to this toxic substance in a dose-dependent fashion. This indicates that the inhibition was more important as the amount of NaF added to the media increased, and at the same time, an increase in MDA concentration in the erythrocyte membrane was observed. Based on our findings, we hypothesize that one damage mechanism of NaF is the inhibition of antioxidant enzymes in the erythrocyte. This inhibition of antioxidant enzymes causes an excess of ROS, which damage the membrane of the erythrocyte, causing an increase in MDA concentration, in turn resulting in oxidative stress caused by this toxin. This oxidative stress is partially reversed by the presence of Vit-E.

The in vitro damage caused by NaF is partially reversed by vitamin E, which is a well-known natural antioxidant. Furthermore, there are reports of in vivo experiments where the pretreatment with vitamin E in combination with methionine and L-carnosine prior to the application of NaF resulted in significant reduction of the damage to various organs [41]. This points to the important role they can have as natural antioxidant compounds in preventing the toxic damage of NaF. In this regard, in addition to vitamin E, other well-known natural products (e.g., curcumin, N-acetylcysteine, and Ginkgo biloba), with antioxidant properties, have been used in in vivo and in vitro experiments to prevent or counteract the damage caused by free radicals generated by the intoxication with NaF and other toxic compounds that frequently contaminate the environment, mainly aquifers [41–45].

Our results show that the presence of Vit-E partially prevented the inhibition of the activity of antioxidant enzymes caused by NaF. We hypothesize that Vit-E may protect the erythrocyte membrane by “trapping” ROS that “attack” it. Nonetheless, the mechanism by which Vit-E prevents the inhibition of SOD, CAT, and GlPx antioxidant enzymes induced by NaF must be investigated in more detail. This could suggest how the damage this toxin causes in enzymatic systems and the cell in general can be avoided because the induction of oxidative stress in cells and hematopoietic tissues may be part of the physiopathological damage mechanism of this toxin.

## 5. Conclusion

In conclusion, the results observed in this study demonstrated that in human erythrocytes incubated in vitro in the presence of NaF, there was an increase in MDA concentration in the membrane and significant deterioration in the activity of the antioxidant enzymes SOD, CAT, and GlPx. This deterioration caused by NaF was partially reverted by the presence of Vit-E, a well-known natural antioxidant.
